# Vitamin D Deficiency Prevalence and Predictors in Early Pregnancy among Arab Women

**DOI:** 10.3390/nu10040489

**Published:** 2018-04-15

**Authors:** Sara Al-Musharaf, Mona A. Fouda, Iqbal Z. Turkestani, Abdulrahman Al-Ajlan, Shaun Sabico, Abdullah M. Alnaami, Kaiser Wani, Syed Danish Hussain, Buthaynah Alraqebah, Amal Al-Serehi, Naemah M. Alshingetti, Nasser Al-Daghri, Philip G McTernan, Sunil J. Wimalawansa, Ponnusamy Saravanan

**Affiliations:** 1 Division of Health Sciences, Warwick Medical School, University of Warwick, Coventry CV4 7AL, UK; sara.almosharruf@gmail.com (S.A.-M.); eaglescout01@yahoo.com (S.S.); philip.mcternan@ntu.ac.uk (P.G.M.); p.saravanan@warwick.ac.uk (P.S.); 2 Department of Medicine, Endocrinology Division, College of Medicine, King Saud University, Riyadh 11461, Saudi Arabia; monafoudaneel@yahoo.com; 3 Department of Obstetrics and Gynaecology, College of Medicine, King Saud University, Riyadh 11461, Saudi Arabia; iqbalzmt@hotmail.com; 4 Department of Clinical Lab Sciences, College of Applied Medical Sciences, King Saud University, Riyadh 11451, Saudi Arabia; aalajl@hotmail.com; 5 Prince Mutaib Chair for Biomarkers of Osteoporosis, Department of Biochemistry, College of Science, King Saud University, Riyadh 11451, Saudi Arabia; aalnaami@yahoo.com (A.M.A.); wani.kaiser@gmail.com (K.W.); danishhussain121@gmail.com (S.D.H.); Buthinah.ali@gmail.com (B.A.); 6 Maternal-Fetal Medicine Department, King Fahad Medical City, Riyadh 59406, Saudi Arabia; aalserehi@kfmc.med.sa; 7 Obstetrics and Gynaecology Department, King Salman Bin Abdulaziz Hospital, Riyadh 11564, Saudi Arabia; alshingetti@yahoo.com; 8 Department of Biosciences, School of Science and Technology, Nottingham Trent University, Nottingham NG11 8NS, UK; 9 Department of Medicine, Endocrinology & Nutrition, Cardio Metabolic Institute, Somerset, NJ 08873, USA; suniljw@hotmail.com

**Keywords:** vitamin D deficiency, pregnancy, vitamin D, Saudi women

## Abstract

Data regarding the prevalence and predictors of vitamin D deficiency during early pregnancy are limited. This study aims to fill this gap. A total of 578 Saudi women in their 1^st^ trimester of pregnancy were recruited between January 2014 and December 2015 from three tertiary care antenatal clinics in Riyadh, Saudi Arabia. Information collected includes socio-economic, anthropometric, and biochemical data, including serum vitamin D (25(OH)D) levels, intake of calcium and vitamin D, physical activity, and sun exposure indices. Pregnant women with 25(OH)D levels <50 nmol/L were considered vitamin D deficient. The majority of participants (*n* = 468 (81%)) were vitamin D deficient. High levels of indoor activity, whole body clothing, multiparity, total cholesterol/HDL ratio(>3.5), low HDL-cholesterol, and living in West Riyadh were significant independent predictors for vitamin D deficiency, with odds ratios (ORs) (95% confidence interval) of 25.4 (5.5–117.3), 17.8 (2.3–138.5), 4.0 (1.7–9.5), 3.3 (1.4–7.9), 2.8 (1.2–6.4), and 2.0 (1.1–3.5), respectively. Factors like increased physical activity, sun exposure at noon, sunrise or sunset, high educational status, and residence in North Riyadh were protective against vitamin D deficiency with ORs 0.2 (0.1–0.5); 0.2 (0.1–0.6); 0.3 (0.1–0.9); and 0.4 (0.2–0.8), respectively. All ORs were adjusted for age, BMI, sun exposure, parity, summer season, vitamin D intake, multivitamin intake, physical activity, education, employment, living in the north, and coverage with clothing. In conclusion, the prevalence of vitamin D deficiency among Saudi women during early pregnancy was high (81%). Timely detection and appropriate supplementation with adequate amounts of vitamin D should reduce the risks of vitamin D deficiency and its complications during pregnancy.

## 1. Introduction

Vitamin D (25(OH)D) deficiency is a global health problem that increases the risk and severity of many diseases. Over two billion people suffer from vitamin D deficiency (25(OH)D < 50 nmol/L) and this has been implicated in exacerbating a wide variety of conditions, such as osteoporosis, rickets/osteomalacia, autoimmune diseases, cardiovascular diseases, cancer, obesity, and type 2 diabetes mellitus. Vitamin D deficiency also increases the risks of maternal and fetal complications [1,2].

It has been observed in at least one study that majority of Saudi women have sub-optimal levels of vitamin D during their reproductive phase of life [3]. This vitamin D deficiency in Saudi women is further impacted during pregnancy itself, with a noted deteriorating vitamin D status, which may arise in part due to the increased nutritional demands of the growing fetus [4]. This decline in vitamin D levels is also common in Arab Gulf countries, where 10–80% of mothers and as much as 40–80% of their neonates, at the time of delivery, have sub-optimal serum levels of 25(OH)D, highlighting that in the Gulf states the answer may be more complicated than sunlight exposure alone [5,6].

It appears that the etiology of vitamin D deficiency during pregnancy is multi-factorial. The factors that affect this include limited sunlight exposure, but also reduced dietary vitamin D and calcium intake, ethnicity, age, socio-economic status, smoking, repeated pregnancies, obesity, malabsorption syndromes, medications that increase vitamin D catabolism, and chronic liver and/or kidney disorders [7,8,9]. In addition, an individual’s lifestyle has also been shown to influence vitamin D status, such as the amount of exercise, reduced vitamin D intake in the diet, biochemical indices such as triglyceride levels, factors associated with vitamin D deficiency, and prevalence in pregnant Arab women [10,11,12].

However, despite the influence vitamin D deficiency has on pregnancy, few studies have addressed the influence this status has in the Gulf State countries [10,13,14]. Prior studies on Vitamin D deficiency during pregnancy based in the Gulf States have tended to investigate deficiency (25(OH)D < 50 nmol/L) [9] in the late stages of pregnancy, but do not necessarily combine lifestyle factors, biochemical factors, and clinical history sufficiently in a large cohort of Arab women. Prior studies have focused on late stages to determine the impact on maternal and neonatal outcomes, but have been unable to examine factors in early pregnancy that may increase disease risk [10,13,14].

The aims of the current study were, therefore, to identify the prevalence of vitamin D deficiency in Arab women during early pregnancy as well as factors that may contribute to vitamin D deficiency.

## 2. Materials and Methods

### 2.1. Study Population

We randomly selected healthy Saudi pregnant women in their first trimester for this observational study. The sample size was estimated and, assuming a vitamin D deficiency prevalence of 80% [14] at a 5% α level of significance and with ±5% level of precision, 246 participants were needed. To account for the potential dropouts and to keep the statistical power intact, we opted to enroll 578 subjects for this study.

Data were collected between January 2014 and December 2015. The season of the sampling was recorded at both visits. For an analysis of season, in relation to vitamin D status, the months of the year were divided into two periods: April to October was classified as summer, and November to March as winter [15]. The maximum ultraviolet B (UVB) radiation in the kingdom of Saudi Arabia (KSA) occurs in July and the minimum values occur in December [16].

All participants were of Saudi descent and non-smokers. At the time of inclusion, they were at less than 16 weeks of gestation and were non-diabetic. All participants had singleton pregnancies. Those with metabolic or chronic inflammatory disorders, or receiving long-term therapy for any chronic condition, were excluded from the study. Furthermore, those participants taking vitamin D supplementation were also excluded from the study. A total of 578 participants from three tertiary care hospitals in Riyadh (King Fahad Medical City Hospital, King Khalid University Hospital, and King Salman Hospital) met the inclusion criteria. All study participants signed the consent form.

Fasting blood samples from each subject were collected, aliquoted, delivered, and stored at −70 °C at the Biomarkers Research Program (BRP), King Saud University (KSU), Riyadh, KSA, for later biochemical analyses, as one batch. Ethical approval for this study was obtained from the Institutional Review Board (IRB) of the participating hospitals before the commencement of the study.

### 2.2. Assessment of Circulating 25(OH)D and Biochemical Parameters

Serum glucose, calcium, creatinine, phosphorus, and lipids (HDL-cholesterol, total cholesterol, and triglycerides (TG)) were measured by using the routine laboratory analysis (Konelab, Espoo, Finland). The instrument was routinely calibrated with quality control samples. LDL-cholesterol and total cholesterol to HDL ratio were calculated using previously published equations [17]. The cut off value for serum triglycerides was ≥1.7 mmol/L, and an HDL-cholesterol <1.03 mmol/L was considered low [18,19]. The BRP laboratory participates in external quality control programs.

Bone alkaline phosphatase was measured using the LIAISON XL immunoassay (DiaSorin, Saluggia VC, Italy). The HbA1c in whole blood was measured using the DCA Vantage Analyzer (Siemens Healthcare, Erlangen, Germany) point-of-care device. Total 25(OH)D and serum insulin were measured with an electro-chemiluminescence binding assay (ECLIA) (Cobase-E-411; Roche Diagnostics GmbA, Mannheim, Germany). The repeatability and intermediate coefficients of variation (CV) for the 25(OH)D assay were 4.4% and 6.6%, respectively, with 100% cross-reactivity to 25(OH)D3 and 92% cross-reactivity to 25(OH)D2. The measurement range is between 7.5–175 nmol/L (3.0–70.0 mg/L). The BRP laboratory participates in the Vitamin D External Quality Assessment Scheme (DEQAS). Vitamin D status was categorized as 25(OH)D < 25 nmol/L as severe deficiency, 25–49.9 nmol/L as deficiency, 50.0–74.9 nmol/L as sufficiency, and ≥75 nmol/L was considered as desirable [9].

### 2.3. Clinical Assessment

Anthropometrics included pre-pregnancy weight (as recalled by the subject), current weight (kg), height (cm), waist (cm), and hip circumferences (cm). Weight and height were recorded using an international standard scale (Digital Pearson Scale, ADAM Equipment Inc., Oxford, CT, USA). Body mass index (BMI) and waist to hip ratio were calculated as described previously [20]. Triceps, biceps, supra iliac, and subscapular thickness were also measured. Relative body fat percentage (%) was estimated by measuring subcutaneous skinfold thickness with a Harpenden caliper (British Indicators, Sussex, Brighton, England) following the protocol described by Durnin and Womersly [21].

### 2.4. Data Collection (Questionnaire), Maternal Hypertension and Assessment of Physical Activity

Gestational age was calculated from the date of the last menstrual period and was confirmed using an ultrasound examination during the first visit. All participants answered a generalized questionnaire [22], which included information on socio-demographics, parity, past medical history, medication treatment history, and sun-exposure. Education was categorized into two groups (High school and below and College/University) and employment as either housewife or employed. Information on sun exposure (yes/no) and other sun exposure indices, such as exposure to the sun at work (indoor or outdoor work), timing of the exposure (noon alone, or with either sunrise or sunset vs. just sunset or sunrise), clothing (whole body coverage vs. some parts of the body exposed), and the use of sunscreen (yes/no), were collected. Noon time was defined as 10:00 a.m. to 2:00 p.m.

A food frequency questionnaire (FFQ), specific for vitamin D and calcium intake, was measured over a period of 7-days in this study, similar to other Saudi studies [23]. The FFQ was designed to calculate vitamin D and calcium intake from different food sources and has been pilot-tested in 60 pregnant women. The assessment of everyday food intake was conducted to record participants’ total intake of macro- and micro-nutrients, especially vitamin D and calcium. Nutrient intake was calculated using the Nutribase software [24] and another table published for Saudi foods [25] was also used. Data on supplement intake (yes/no) were collected, including multivitamins, folic acid, iron, and cod liver oil. Subjects on vitamin D supplementation were excluded from the study. Gestational hypertension was defined as blood pressure ≥ 140/90 mmHg, based on standards from the Int. Society for the Study of Hypertension in Pregnancy [26].

The international physical activity questionnaire (IPAQ) was used to assesses the level of physical activity [27], using its Arabic version. Low-intensity physical activity (e.g., walking and sitting time) was determined using the guidelines for data processing and analysis of the IPAQ [28]. Less than 210 min per week was identified as low-intensity physical activity [29].

### 2.5. Data Analysis

Data were analyzed using SPSS version 21.0 statistical software (SPSS, Chicago, IL, USA). Normality was tested before performing the analysis using the Shapiro-Wilk Test. Quantitative normal variables were expressed as mean and standard deviation, while quantitative non-normal variables were expressed as medians. Categorical variables were expressed as frequencies and percentages (%). Pearson’s Chi-square test was used to determine the association between categorical variables. Comparisons between groups were performed using the Independent *t*-test and Mann-Whitney U test for normal and non-normal variables respectively.

Logistic regression was used to identify risk factors associated with vitamin D deficiency. Confounders included in the model were age, BMI, sun exposure, parity, season, vitamin D intake, multivitamin intake, physical activity, education, employment, location (i.e., living in North Riyadh vs. South Riyadh), and the extent of skin coverage with clothing. All odd ratios (ORs) were reported (univariate and adjusted model). A *p*-value of <0.05 was considered statistically significant.

## 3. Results

### 3.1. Subject Characteristics by Vitamin D Status

#### 3.1.1. General Characteristics of Participants during First Trimester of Pregnancy

A total of 578 subjects were included in this study. The mean maternal age was 28.8 ± 5.4 (range 18–39) years. Among them, 320 (57.3%) were university graduates or post-graduates, 183 (32.7%) were employed, and 62 (12%) were earning more than SAR 10,000/month. One-fourth of these women (28.4%) was residing in West Riyadh, with comparable percentages living in the south (21.9%), east (20.5%), and north (19%) of the city. Among participants, 129 (30.5%) had a history of caesarean section and 120 (29.6%) had previous miscarriages. Thirty-eight (8.8%) suffered from gestational diabetes mellitus (GDM) in the past (but not at the time enrolled into the present study), 164 (38.2%) had a family history of GDM, and 347 (72.6%) had a family history of diabetes. The rest are shown in Table 1.

#### 3.1.2. Prevalence of Vitamin D deficiency in Early Pregnancy

The median (1^st^–3^rd^ quartile) 25(OH)D concentration in the sample population was 28.7 nmol/L (19.7–43.8). Severe vitamin D deficiency was noted in 41.7% (241/578) of participants, 39.3% (227/578) were deficient, and 13.7% (79/578) had insufficiency in circulating vitamin D. Only 5.4% (31/578) of participants presented with the physiological normal levels of 25(OH)D. A small number of participants (*n* = 31; 5.4%) had desirable vitamin D levels (≥75 nmol/L). Participants’ 25(OH)D levels were categorized into deficient (<50 nmol/L) or non-deficient (≥50 nmol/L) groups for meaningful statistical analysis [9]. The proportion of vitamin D deficiency (<50 nmol/L) in early pregnancy amounted to 81% (468/578).

#### 3.1.3. Vitamin D Status among Various Socio-Economic Groups

Socioeconomic data were presented in valid percentages as not all subjects chose to answer certain demographic variables. Subjects with graduate- or postgraduate-level education were more likely to be non-deficient compared to ones with high school or lesser education (71.3% vs. 54%, *p* < 0.001). Housewives were more likely to be vitamin D deficient compared to employed women (56.5% vs. 43.5%, *p* = 0.008).

In West Riyadh, a significantly higher percentage of pregnant women (30.8%) were in the vitamin D deficient group compared to 18.4% in the non-deficient group (*p* = 0.014). In contrast, in North Riyadh, a significantly higher percentage (30.6%) was in the non-deficient group compared to 16.2% in the vitamin D deficient group (*p* = 0.001). Furthermore, the pregnant women living in North Riyadh were significantly more educated, at least to the graduate level (*p* = 0.001), compared with their counterparts residing in West Riyadh (*p* = 0.002) (Table 1).

### 3.2. Anthropometric and Biochemical Characteristic in Relation to Vitamin D Status

Gestational age was significantly higher in the deficient than the non-deficient group, even after adjustments for BMI and age (*p* = 0.04). Body fat percentage was significantly higher in the non-deficient group compared to the deficient group (35.4 ± 5.1 vs. 34.1 ± 5.6, *p* = 0.04) (Table 2).

HDL-cholesterol was significantly lower in the deficient group than the non-deficient group (1.4 ± 0.3 vs. 1.3 ± 0.4, *p* = 0.035) and remained significant after age and BMI adjustments. Total cholesterol/HDL ratio was significantly higher in the deficient group than the non-deficient group (*p* < 0.001). Vitamin D status was significantly and positively correlated with HDL-cholesterol (*r* = 0.19, *p* < 0.001), while an inverse significant correlation was observed between 25(OH)D levels and total cholesterol/HDL ratio, random glucose, and HbA1c (*r* = −0.15, *p* < 0.001; *r* = −0.11, *p* = 0.008; *r* = −0.13, *p* = 0.001), respectively (Table 2).

### 3.3. Lifestyle Factors in Relation to Vitamin D Status

The preferable timing of the sun exposure, nature of work, and cloth coverage, all had a significant effect on vitamin D status. Of the pregnant women in the non-deficient group 64.5% preferred sun-exposure at noon time, while 79.9% of the ones in the deficient group preferred it at sunrise or sunset (*p* < 0.001). The proportion of pregnant women involved in indoor activities for a longer duration was higher in the vitamin D deficient group compared to the non-deficient group (97.6% vs. 67.3%, *p* < 0.001). Similarly, a significantly higher proportion of pregnant women who usually cover their whole body were in the vitamin D deficient group than the non-deficient group (38.9% vs. 3.6%, *p* < 0.001).

Physical activity also had a significant effect on vitamin D status. The median number of minutes/week for women engaged in low-intensity physical activity (e.g., walking) was significantly higher in the non-deficient than in the deficient group, even after adjusting for age and BMI (600 vs. 180 min/week, *p* < 0.001). Physical activity correlated positively with levels of vitamin D (*r* = 0.32, *p* < 0.001). Dietary vitamin D and calcium intake, as well as multivitamin intake, were not significantly different from one another (Table 3).

### 3.4. Predictors of Vitamin D Deficiency in Early Pregnancy

Table 4 highlights the predictors of vitamin D deficiency in early pregnancy. Significant risk factors for vitamin D deficiency were indoor nature of work (OR 25.4, 95% CI 5.5–117.3, *p* < 0.001), whole body coverage with clothing (OR17.8, 95% CI 2.3–138.5, *p* = 0.006); multiparity (OR 3.97, 95% CI 1.7–9.5, *p* = 0.002), total cholesterol/HDL ratio (>3.5) (OR 3.3, 95% CI 1.4–7.9, *p* = 0.007); low HDL-cholesterol (OR 2.8, 95% CI 1.2–6.4, *p* = 0.015), and living in West Riyadh (OR 2.0, 95% CI 1.1–3.5, *p* = 0.011). Factors that conferred protection against vitamin D deficiency in early pregnancy were low-intensity physical activity (OR 0.20, 95% CI 0.09–0.47, *p* < 0.001), sun exposure at noon (OR 0.24, 95% CI 0.10–0.57, *p* = 0.001), educational status of graduate or higher (OR 0.32, 95% CI 0.12–0.86, *p* = 0.02); residence in North Riyadh (OR 0.35, 95% CI 0. 15–0.79, *p* = 0.012), and age (OR 0.91, 95% CI 0.83–1.00, *p* = 0.05).

## 4. Discussion

To the best of our knowledge, this study has evaluated the largest number of participants, within Saudi Arabia and the Gulf region, assessing vitamin D deficiency in the early stages of pregnancy along with the predictors of vitamin D deficiency. In this study, the prevalence of vitamin D deficiency (vitamin D < 50 nmol/L) among pregnant women in their first trimester was 81%, with noted significant predictors for vitamin D deficiency including multiparity, living in West Riyadh, abnormal lipids, indoor nature of activity, and type of clothing. Furthermore, higher education, living in North Riyadh, sun exposure at noon, and physical activity were noted as protective against vitamin D deficiency. Despite the high prevalence of vitamin D deficiency, it remains comparable with the findings of previous studies published on Asian populations [30,31]. Notably a recent study, also conducted in Riyadh, investigating vitamin D status among pregnant women, detected vitamin D deficiency at 50% and insufficiency as 43.8% [10]. Even higher prevalence rates of vitamin D deficiency during early pregnancy have been reported from Oman (98%) [11], Korea (91.8%) [32], and UAE (78%) [33]. Comparatively lower prevalence rates for vitamin D deficiency during early pregnancy have also been reported from India (62%) [34], UK (57%) [35], Canada (39%) [36], and Spain (18%) [37]. This high prevalence globally of vitamin D deficiency in pregnant women still stresses the importance of vitamin D supplementation in early pregnancy (<16 weeks gestation).

In this study, inadequate exposure to sunlight was observed to be a major risk factor for suboptimal vitamin D levels. Sunlight exposure, being a major source of vitamin D, is critical in maintaining normal 25(OH)D levels [38]. Limited exposure to sunlight is, therefore, considered to be an important predictor of vitamin D deficiency even in places considered to have high levels of sunshine [38]. Spending a significant amount of time indoors to avoid sun exposure, clothing, and the use of sun cream have been recognized as major risk factors for developing vitamin D deficiency [39].

The pregnant women in the present study were noted to have inadequate sun exposure due to extreme weather temperatures as a result of the low humidity in desert environments (summer vs. winter) and the traditional clothing they wear, which covers the whole body. Whole body coverage poses a 17-times higher risk for developing vitamin D deficiency, which is applicable to most Muslim countries [40]. These factors would appear to have contributed to the observed high prevalence of vitamin D deficiency in this current study.

Despite this, seasonal variations, here operationalized as recruitment in summer or winter, did not significantly affect the distribution of the two vitamin D groups. However, even though the season would appear to dictate the amount of available sunlight, this lack of effect was anticipated due to coverage of the whole body with clothing, sun-avoidance during summer months due to heat, and a large proportion of the work being performed indoors in Saudi Arabia. Consequently, the exposure indices are unaffected by season amongst the Saudi women.

Approximately 25% of the pregnant women were taking multivitamins in their first trimester. A study by Hollis et al. suggested that daily supplementation of 4000 IU/day of vitamin D is considered to be safe, and reasonably adequate for avoiding vitamin D deficiency in pregnancy [41]. However, vitamin D intake from food was observed to be low in the participant sample studied here, with very few subjects consuming the daily recommended intake. The major source of vitamin D is the cutaneous synthesis from sun exposure, as dietary sources tend to be small despite any fortification of food. Reliance solely on dietary sources is known to lead to hypovitaminosis D [41]. Moreover, recent food trends and changes in eating habits in Saudi Arabia may also have contributed to a lower dietary intake of vitamin D and calcium, especially among younger women [42]. Hence, suggesting a country-wide vitamin D fortification of food, especially grain and dairy products might be helpful.

In addition, multiparity and successive unplanned pregnancies in a community with a high prevalence of vitamin D deficiency may further aggravate the condition [5]. Pregnant housewives with low educational status, residing in low-income localities, are strong indicators of vulnerability to vitamin D deficiency, which is in part due to a lack of awareness in this particular group. This observation has previously been reported in a study documenting higher vitamin D levels among pregnant women with higher educational status compared to less educated pregnant women [43].

Further analysis revealed physical activity as an independent predictor of 25(OH)D deficiency among pregnant women. Studies performed among non-pregnant female populations confirmed the beneficial effect of physical activity on vitamin D status, after adjusting for sunlight exposure [44]. In addition, exercise-induced lipolysis has also been implicated in the elevation of serum vitamin D levels by mobilizing vitamin D from adipose tissues [45]. However, only a few pregnant women had desirable vitamin D levels, which was associated with increased physical activity outdoors [10,46], thus creating more opportunities for sun exposure, and consequently leading to higher vitamin D levels.

Assessing systemic lipid levels showed that a low HDL and total cholesterol/HDL ratio presented a significant risk factor for vitamin D deficiency in early pregnancy. It has previously been shown that 25(OH)D concentration positively correlates with HDL cholesterol, even after adjusting for age, BMI, ethnicity, family history of diabetes, and cigarette smoking [35]. Studies have also shown that more than half of the dietary intake of vitamin D comes from fish, meat, and eggs, which are also rich in lipids [47]. In addition to the association of hypovitaminosis D with reduced HDL-cholesterol levels, this combination could further increase the risks for the development of cardiovascular diseases [48].

The authors acknowledge several limitations of this study. First is the FFQ-based dietary vitamin D intake, which does not reliably quantify 25(OH)D in episodically consumed foods. Vitamin D as a metabolite is influenced by diet and as such, FFQ might not be the best option to determine an accurate dietary vitamin D assessment. Second is the limitations of the immunoassays used. Though all 25(OH)D measurements were conducted in a DEQAS-certified laboratory, it is still preferable to use a VDSP (Vitamin D Standardization Program), as it is an accuracy-based scheme. Despite the abundance of data on vitamin D deficiency and pregnancy, the present study offers insights on unidentified risk factors associated with vitamin D deficiency in early pregnancy in an Arabian cohort, an ethnic population that is geographically, culturally, and possibly genetically predisposed to vitamin D deficiency.

## 5. Conclusions

In conclusion, there is a high prevalence of vitamin D deficiency among Saudi pregnant women during the first trimester of pregnancy. Whole body clothing coverage, a low level of physical activity, living in West Riyadh, and multi-parity were key factors pre-disposing Saudi pregnant women to increased vitamin D deficiency.

The findings of the present study highlight the need for vitamin D supplementation among women during their reproductive age, especially women entering pregnancy. Further studies are needed to determine whether untreated vitamin D deficiency during early pregnancy translates to maternal and neonatal complications among Arab women.

## Figures and Tables

**Table 1:** Subject characteristics by vitamin D status in early pregnancy.

	Total (*n* = 578)	Non-Deficient (*n* = 110)	Deficient (*n* = 468)	*p*-Values
25(OH)D (nmol/L)	28.7 (19.7–43.8)	64.5 (57.0–76.6)	24.4 (18.0–33.8)	<0.001
Sociodemographics				
**Education**				
High School or Lower	42.7 (238)	28.7 (31)	46 (207)	0.001
College/University level	57.3 (320)	71.3 (77)	54 (243)	
**Income**				
<10,000 Saudi Riyal	88 (454)	86.5 (83)	88.3 (371)	0.61
≥10,000 Saudi Riyal	12 (62)	13.5(13)	11.7 (49)	
**Living area**				
North Riyadh	19 (98)	30.6 (30)	16.2 (68)	0.018
West Riyadh	28.4 (147)	18.4 (18)	30.8 (129)	
East Riyadh	20.5 (106)	19.4 (19)	20.8 (87)	
South Riyadh	21.9 (113)	20.4 (20)	22.2 (93)	
Center Riyadh	9.2 (53)	10 (11)	9 (42)	
**Employment**				
Housewife	67.3 (376)	56.5 (61)	69.8 (315)	0.008
Employed	32.7 (183)	43.5(47)	30.2 (136)	
Obstetric and family history				
**Multiparous**	56.7 (328)	11.3 (53)	2.5 (275)	0.11
Caesarean section	30.5 (129)	36.3 (29)	29.2 (100)	0.21
Miscarriage	29.6 (120)	31.1 (23)	29.3 (97)	0.76
Family history of DM	72.6 (347)	74.2 (66)	72.2 (281)	0.71
Family history of obesity	16.8 (65)	22.4 (15)	15.6 (50)	0.17

Note: Categorical variables are presented as valid percentages (*n*); DM - diabetes mellitus.

**Table 2:** Anthropometric and biochemical parameters in relation to vitamin D status in early pregnancy.

	Total (*n* = 578)	Non-Deficient (*n* = 110)	Deficient (*n* = 468)	*p*-Values
25(OH)D (nmol/L) #	28.7 (19.7–43.8)	64.5 (57.0–76.6)	24.4 (18.0–33.8)	<0.001
Anthropometric Parameters				
Age (years)	28.8 ± 5.4	29.4 ± 5.2	28.6 ± 5.5	0.19
Gestational Age (Weeks)	12.0 ± 3.0	11.5 ± 2.9	12.1 ± 3.0	0.066
Pre-preg. BMI (kg/m^2^)	27.0 ± 6.0	27.1 ± 6.2	27.0 ± 5.9	0.944
BMI (kg/m^2^)	28.0 ± 6.3	28.3 ± 6.3	28.0 ± 6.3	0.575
Waist (cm)	91.5 ± 13.6	93.1 ± 12.4	91.1 ± 13.8	0.18
Hips (cm)	107.9 ± 12.0	108.8 ± 11.6	107.7 ± 12.1	0.37
Waist-Hip Ratio	0.8 ± 0.1	0.9 ± 0.1	0.8 ± 0.1	0.34
Systolic BP (mm Hg)	113.9 ± 12.7	114.3 ± 13.3	113.8 ± 12.6	0.70
Diastolic BP(mm Hg)	67.9 ± 9.6	67.1 ± 9.8	68.0 ± 9.6	0.38
Body Fat (%)	34.3 ± 5.6	35.4 ± 5.1	34.1 ± 5.6	0.03
Biochemical Parameters				
Calcium (mmol/L)	2.2 ± 0.2	2.3 ± 0.2	2.2 ± 0.2	0.44
Phosphorus (mmol/L)	1.2 ± 0.4	1.2 ± 0.5	1.1 ± 0.4	0.13
Alkaline Phos. (mmol/L)	9.7 ± 3.3	9.3 ± 2.9	9.7 ± 3.4	0.27
Creatinine (µmol/L)	55.8 ± 18.2	55.4 ± 17.9	55.9 ± 18.3	0.81
Glucose (mmol/L) #	4.8 (4.4–5.3)	4.7 (4.4–5.2)	4.8 (4.4–5.3)	0.63
Insulin (uU/mL) #	8.5 (4.8–18.4)	7.9 (4.6–17.7)	8.7 (4.9–18.4)	0.43
HbA1c	5.1 ± 0.5	5.1 ± 0.5	5.1 ± 0.5	0.49
Total Cholesterol (mmol/L)	5.2 ± 1.0	5.2 ± 0.8	5.2 ± 1.0	0.75
HDL-Cholesterol (mmol/L)	1.3 ± 0.3	1.4 ± 0.3	1.3 ± 0.4	0.035
Total Chol-HDL Ratio	4.0 ± 1.0	3.8 ± 0.7	4.1 ± 1.1	<0.001
LDL-Cholesterol (mmol/L)	3.2 ± 0.8	3.1 ± 0.6	3.2 ± 0.8	0.68
Triglycerides (mmol/L)	1.4 ± 0.6	1.4 ± 0.6	1.4 ± 0.6	0.94

Note: Data presented as a mean ± standard deviation for normal variables while median (first quartile, third quartile) were presented for non-normally distributed variables. # indicates non-normally distributed variables. Categorical variables were presented as percentages (*n*). The *p*-value for mean differences was obtained from an independent sample *t*-test for normal variables and Mann-Whitney U test for non-normally distributed variables. Significant at *p* < 0.05.

**Table 3:** Life-style factors in relation to vitamin D status in early pregnancy.

	Total )*n* = 578(	Non-Deficient (*n* = 110)	Deficient (*n* = 468)	*p*-Values
25(OH)D (nmol/L) #	28.7 (19.7–43.8)	64.5 (57.0–76.6)	24.4 (18.0–33.8)	<0.001
Life-style factors				
**Season**				
Summer	39.4 (225)	41.5 (44)	38.9 (181)	0.62
Winter	60.6 (346)	58.5 (62)	61.1 (284)	
**Time of sun exposure**				
noon time	28.5 (165)	64.5 (71)	20.1 (94)	**<0.001**
Sun set or sunrise	71.4 (413)	35.5 (39)	79.9 (374)	
**Nature of work**				
Indoor	91.9 (531)	67.3 (74)	97.6 (457)	**<0.001**
Outdoor	81 (47)	32.7 (36)	2.4 (11)	
**Cloth coverage**				
Whole body coverage	32.2 (186)	3.6 (4)	38.9 (182)	**<0.001**
Face or hand or feet exposed	67.8 (392)	96.4 (106)	61.1 (286)	
**Usage of sunscreen (Yes)**	6.2 (36)	10 (11)	5.3 (25)	0.07
**Vitamin D intake (IU/day) #**	89.9 (63.5–169)	89.3 (61.3–127.5)	95.6 (64.9–180)	0.36
**Calcium intake (mg/day) #**	117.7 (60.2–370.3)	118.5 (67.5–229.9)	117.4(59.9–400)	0.99
**Multivitamin**	25(100)	24.1 (27)	28.6 (134)	0.42
**Physical Activity (min/week) #**	210 (70–600)	600 (180–1200)	180 (60–420)	**<0.001**

Note: Categorical variables are presented as percentages (*n*). # indicates non-normally distributed variables which are presented as median (first quartile, third quartile).

**Table 4:** Predictors of vitamin D deficiency among pregnant women in early pregnancy.

Parameters	Univariate Analysis		Adjusted Model	
	OR (95% CI)	*p*-Value	OR (95% CI)	*p*-Value
Age (years)	0.97 (0.94–1.01)	0.19	0.91 (0.83–1.00)	0.05
Gestational age	1.07 (0.99–1.15)	0.07	0.99 (0.86–1.13)	0.88
Multiparity	1.56 (1.00–2.43)	0.05	3.97 (1.66–9.48)	**0.002**
Pre-pregnancy BMI (kg/m^2^)	1.00 (0.96–1.03)	0.94	0.99 (0.92–1.06)	0.73
Obesity by 1st visit	0.86 (0.55–1.35)	0.52	0.95 (0.41–2.19)	0.91
University graduate or postgraduate	0.47 (0.30–0.75)	0.001	0.32 (0.12–0.86)	**0.023**
Employment	0.56 (0.36–86)	0.008	0.61 (0.27–1.34)	0.60
Living in North Riyadh	0.44 (0.27–0.73)	0.001	0.35 (0.15–0.79)	**0.012**
Living in West Riyadh	1.41 (1.07–1.85)	0.01	2.00 (1.14–3.53)	**0.016**
HbA1c at 1st visit	1.14 (0.78–1.68)	0.49	1.34 (0.62–2.90)	0.46
Random glucose (mmol/L) at 1st visit	1.02 (0.85–1.23)	0.63	0.84 (0.61–1.17)	0.30
Triglycerides (≥1.7 mmol/L)	1.06 (0.67–1.69)	0.80	0.62 (0.24–1.59)	0.62
Total cholesterol/HDL ratio (>3.5)	1.37 (0.89–2.11)	0.16	3.30 (1.38–7.90)	**0.007**
Low HDL-cholesterol (<1.03 mmol/L)	1.66 (1.09–2.54)	0.02	2.81 (1.22–6.42)	**0.015**
Hypertension	0.86 (0.31–2.36)	0.77	5.1(0.23–110.38)	0.30
Sun exposure at noon time	0.14 (0.09–0.22)	<0.001	0.24 (0.10–0.57)	**0.001**
Indoor nature of work	20.2 (9.8–41.46)	<0.001	25.4 (5.5–117.3)	**<0.001**
Clothing (whole body cover)	16.9 (6.1–46.55)	<0.001	17.8 (2.3–138.5)	**0.006**
Vitamin D intake (>600 IU/day)	0.35 (0.11–1.12)	0.06	0.35 (0.07–1.93)	0.23
Calcium Intake (>1000 mg/day)	0.62 (0.23–1.64)	0.33	0.86 (0.14–5.14)	0.87
Use of multivitamin supplements	0.80 (0.46–1.39)	0.42	0.62 (0.25–1.54)	0.30
Physical Activity (≥210 min/week)	0.28 (0.17–0.47)	<0.001	0.20 (0.09–0.47)	**<0.001**

Note: Odds ratio (OR) and 95% CI for OR were obtained using logistic regression analysis, taking vitamin D deficiency (<50 nmol/L) as a dependent variable against potential risk factors, and as an independent risk. Model 1 is for age, BMI, and sun exposure. The adjusted model includes age, BMI, sun exposure, parity, summer season, vitamin D intake, multivitamin intake, physical activity, education, employment, living in the north, and coverage with clothing. Significance is set at *p* < 0.05.

## References

[1] Hilger J., Friedel A., Herr R., Rausch T., Roos F., Wahl D.A., Pierroz D.D., Weber P., Hoffmann K. (2014). A systematic review of vitamin D status in populations worldwide. Br. J. Nutr..

[2] Aghajafari F., Nagulesapillai T., Ronksley P.E., Tough S.C., O’Beirne M., Rabi D.M. (2013). Association between maternal serum 25-hydroxyvitamin D level and pregnancy and neonatal outcomes: Systematic review and meta-analysis of observational studies. BMJ.

[3] Fouda M.A., Turkistani I.Z., Angkaya-Bagayawa F.F., Krishnaswamy S., Al-Daghri N. (2016). Vitamin D deficiency in young women of childbearing age: The elephant in the room. Int. J. Clin. Exp. Med..

[4] Urrutia R.P., Thorp J.M. (2012). Vitamin D in pregnancy: Current concepts. Curr. Opin. Obstet. Gynecol..

[5] Aly Y.F., El Koumi M.A., El Rahman R.N.A. (2013). Impact of maternal vitamin D status during pregnancy on the prevalence of neonatal vitamin D deficiency. Pediatr. Rep..

[6] Kazemi A., Sharifi F., Jafari N., Mousavinasab N. (2009). High prevalence of vitamin D deficiency among pregnant women and their newborns in an Iranian population. J. Womens Health (Larchmt).

[7] Holick M., Chen T. (2008). Vitamin D deficiency: A worldwide problem with health consequences. Am. J. Clin. Nutr..

[8] Karras S., Paschou S.A., Kandaraki E., Anagnostis P., Annweiler C., Tarlatzis B.C., Hollis B.W., Grant W.B., Goulis D.G. (2016). Hypovitaminosis D in pregnancy in the Mediterranean region: A systematic review. Eur. J. Clin. Nutr..

[9] Al-Daghri N.M., Al-Saleh Y., Aljohani N., Sulimani R., Al-Othman A.M., Alfawaz H., Fouda M., Al-Amri F., Shahrani A., Alharbi M. (2017). Vitamin D status correction in Saudi Arabia: An experts’ consensus under the auspices of the European Society for Clinical and Economic Aspects of Osteoporosis, Osteoarthritis and Musculoskeletal Diseases (ESCEO). Arch. Osteoporos..

[10] Al-Faris N.A. (2016). High Prevalence of Vitamin D Deficiency among Pregnant Saudi Women. Nutrients.

[11] Hwalla N., Al Dhaheri A.S., Radwan H., Alfawaz H.A., Fouda M.A., Al-Daghri N.M., Zaghloul S., Blumberg J.B. (2017). The prevalence of micronutrient deficiencies and inadequacies in the Middle East and approaches to interventions. Nutrients.

[12] Al-Ajlan A., Krishnaswamy S., Alokail M.S., Aljohani N.J., Al-Serehi A., Sheshah E., Alshingetti N.M., Fouda M., Turkistani I.Z., Al-Daghri N.M. (2015). Vitamin D deficiency and dyslipidemia in early pregnancy. BMC Pregnancy Childbirth.

[13] Al Kalbani M., Elshafie O., Rawahi M., Al-Mamari A., Al-Zakwani A., Woodhouse N. (2011). Vitamin D status in pregnant Omanis: A disturbingly high proportion of patients with low vitamin D stores. Sultan Qaboos Univ. Med. J..

[14] Azhar W. (2009). A Determination of Vitamin D Status and Intake of Pregnant and Non-Pregnant Saudi Arabian Women in Mecca, Saudi Arabia. Master’s Theses and Doctoral Dissertations.

[15] Al-Daghri N.M., Al-Attas O.S., Alokail M.S., Alkharfy K.M., El-Kholie E., Yousef M., Al-Othman A., Al-Saleh Y., Sabico S., Kumar S. (2012). Increased vitamin D supplementation recommended during summer season in the gulf region: A counterintuitive seasonal effect in vitamin D levels in adult, overweight and obese Middle Eastern residents. Clin. Endocrinol..

[16] Mahfoodh M.B., Al-Ayed M., Al-Dhafiri A. (2003). Measurement and assessment of ultraviolet radiation in Riyadh, Saudi Arabia. Int. J. Sustain. Energy.

[17] Friedewald W.T., Levy R.I., Fredrickson D.S. (1972). Estimation of the concentration of low-density lipoprotein cholesterol in plasma, without use of the preparative ultracentrifuge. Clin. Chem..

[18] Kannel W.B. (1983). High-density lipoproteins: Epidemiologic profile and risks of coronary artery disease. Am. J. Cardiol..

[19] Grundy S.M., Cleeman J.I., Daniels S.R., Donato K.A., Eckel R.H., Franklin B.A., Gordon D.J., Krauss R.M., Savage P.J., Smith S.C. (2005). Diagnosis and management of the metabolic syndrome an American Heart Association/National Heart, Lung, and Blood Institute scientific statement. Circulation.

[20] Wang J., Thornton J.C., Bari S., Williamson B., Gallagher D., Heymsfield S.B., Horlick M., Kotler D., Laferrère B. (2003). Comparisons of waist circumferences measured at 4 sites. Am. J. Clin. Nutr..

[21] Durnin J., Womersley J. (1974). Body fat assessed from total body density and its estimation from skinfold thickness: Measurements on 481 men and women aged from 16 to 72 years. Br. J. Nutr..

[22] Aldesi D.A.D. (2014). A Dietary Interventional Study Moderating Fat Intake in Saudi Subjects with Metabolic Disease. Ph.D. Thesis.

[23] Al-Musharaf S., Al-Othman A., Al-Daghri N.M., Krishnaswamy S., Yusuf D.S., Alkharfy K.M., Al-Saleh Y., Al-Attas O.S., Alokail M.S., Moharram O. (2012). Vitamin D deficiency and calcium intake in reference to increased body mass index in children and adolescents. Eur. J. Pediatr..

[24] (2014). NutriBase 11 Professional.

[25] (2007). Arabic programme 1st version.

[26] Redman C.J.-L., Russell R., Powrie R., Greene M., Camann W. (2010). Hypertension in Pregnancy. De Swiet’s Medical Disorders in Obstetric Practice.

[27] Ebrahimi F., Shariff Z.M., Tabatabaei S.Z., Fathollahi M.S., Mun C.Y., Nazari M. (2015). Relationship between sociodemographics, dietary intake, and physical activity with gestational weight gain among pregnant women in Rafsanjan City, Iran. J. Health Popul. Nutr..

[28] IPAQ Research Committee Guidelines for Data Processing and Analysis of the International Physical Activity Questionnaire (Ipaq)–Short and Long Forms. http://www.ipaq.ki.se/scoring.pdf.

[29] Mudd L.M., Owe K.M., Mottola M.F., Pivarnik J.M. (2013). Health benefits of physical activity during pregnancy: An international perspective. Med. Sci. Sports Exerc..

[30] Song S.J., Zhou L., Si S., Liu J., Zhou J., Feng K., Wu J., Zhang W. (2013). The high prevalence of vitamin D deficiency and its related maternal factors in pregnant women in Beijing. PLoS ONE.

[31] Shibata M., Suzuki A., Sekiya T., Sekiguchi S., Asano S., Udagawa Y., Itoh M. (2011). High prevalence of hypovitaminosis D in pregnant Japanese women with threatened premature delivery. J. Bone Miner. Metab..

[32] Choi R., Kim S., Yoo H., Cho Y.Y., Kim S.W., Chung J.H., Oh S.-Y., Lee S.-Y. (2015). High Prevalence of Vitamin D Deficiency in Pregnant Korean Women: The First Trimester and the Winter Season as Risk Factors for Vitamin D Deficiency. Nutrients.

[33] Narchi H., Kochiyil J., Zayed R., Abdulrazzak W., Agarwal M. (2010). Maternal vitamin D status throughout and after pregnancy. J. Obstet. Gynaecol..

[34] Dasgupta A., Saikia U., Sarma D. (2012). Status of 25 D levels in pregnancy: A study from the North Eastern part of India. Indian J. Endocrinol. Metab..

[35] Makgoba M., Nelson S.M., Savvidou M., Messow C.-M., Nicolaides K., Sattar N. (2011). First-trimester circulating 25-hydroxyvitamin D levels and development of gestational diabetes mellitus. Diabetes Care.

[36] Wei S., Audibert F., Hidiroglou N., Sarafin K., Julien P., Wu Y., Luo Z., Fraser W. (2012). Longitudinal vitamin D status in pregnancy and the risk of pre-eclampsia. BJOG.

[37] Rodriguez A., Santa Marina L., Jimenez A.M., Esplugues A., Ballester F., Espada M., Sunyer J., Morales E. (2016). Vitamin D Status in Pregnancy and Determinants in a Southern European Cohort Study. Paediatr. Perinat. Epidemiol..

[38] Holick M.F. (2004). Sunlight and vitamin D for bone health and prevention of autoimmune diseases, cancers, and cardiovascular disease. Am. J. Clin. Nutr..

[39] Xiang F., Jiang J., Li H., Yuan J., Yang R., Wang Q., Zhang Y. (2013). High prevalence of vitamin D insufficiency in pregnant women working indoors and residing in Guiyang, China. J. Endocrinol. Invest..

[40] Ates S., Sevket O., Ozcan P., Ozkal F., Kaya M.O., Dane B. (2016). Vitamin D status in the first-trimester: Effects of Vitamin D deficiency on pregnancy outcomes. Afr. Health Sci..

[41] Holick M.F., Binkley N.C., Bischoff-Ferrari H.A., Gordon C.M., Hanley D.A., Heaney R.P., Murad M.H., Weaver C.M. (2012). Guidelines for preventing and treating vitamin D deficiency and insufficiency revisited. J. Clin. Endocrinol. Metab..

[42] Al-Faris N.A., Al-Tamimi J.Z., Al-Jobair M.O., Al-Shwaiyat N.M. (2015). Trends of fast food consumption among adolescent and young adult Saudi girls living in Riyadh. Food Nutr. Res..

[43] Fenina H., Chelli D., Ben Fradj M.K., Feki M., Sfar E., Kaabachi N. (2016). Vitamin D Deficiency is Widespread in Tunisian Pregnant Women and Inversely Associated with the Level of Education. Clin. Lab..

[44] Wanner M., Richard A., Martin B., Linseisen J., Rohrmann S. (2015). Associations between objective and self-reported physical activity and vitamin D serum levels in the US population. Cancer Causes Control.

[45] Tzotzas T., Papadopoulou F., Tziomalos K., Karras S., Gastaris K., Perros P., Krassas G. (2010). Rising serum 25-Hydroxy-vitamin D levels after weight loss in obese women correlate with improvement in insulin resistance. J. Clin. Endocrinol. Metab..

[46] Scragg R., Holdaway I., Singh V., Metcalf P., Baker J., Dryson E. (1995). Serum 25-hydroxyvitamin D3 is related to physical activity and ethnicity but not obesity in a multicultural workforce. Aust. N. Z. J. Med..

[47] Kluczynski M.A., Lamonte M.J., Mares J.A., Wactawski-Wende J., Smith A.W., Engelman C.D., Andrews C.A., Snetselaar L.G., Sarto G.E., Millen A.E. (2011). Duration of Physical Activity and Serum 25-hydroxyvitamin D Status of Postmenopausal Women. Ann. Epidemiol..

[48] Barter P., Gotto A.M., LaRosa J.C., Maroni J., Szarek M., Grundy S.M., Kastelein J.J.P., Bittner V., Fruchart J.-C. (2007). HDL Cholesterol, Very Low Levels of LDL Cholesterol, and Cardiovascular Events. N. Engl. J. Med..

